# The Effects of Cryomilling CNTs on the Thermal and Electrical Properties of CNT/PMMA Composites

**DOI:** 10.3390/polym8050169

**Published:** 2016-04-26

**Authors:** Garima Mittal, Kyong Yop Rhee, Soo Jin Park

**Affiliations:** 1 Department of Mechanical Engineering, College of Engineering, Kyung Hee University, Yongin 446-701, Korea; garima.nano@gmail.com; 2 Department of Chemistry, Inha University, Incheon 402-751, Korea

**Keywords:** cryomilled CNT, conductivity, electromagnetic shielding effectiveness, PMMA/CNT composites

## Abstract

In this study, the cryomilling of carbon nanotubes (CNTs) was carried out to accomplish better dispersion without using any hazardous chemicals. Accordingly, different samples of CNTs were prepared by varying the milling speed (10, 20, and 25 Hz) and time (5, 10, and 15 min) and incorporated into the poly(methyl methacrylate) (PMMA) matrix. The changes of the morphology were analyzed by utilizing a field emission scanning electron microscope (FESEM) and a high-resolution transmission electron microscope (TEM). Qualitative analysis of the cryomilled CNTs was carried out using Raman spectroscopy, and their surface area was determined via Brunauer–Emmett–Teller (BET) analysis. Subsequently, thermogravimetric analysis was conducted to evaluate the thermal properties, whereas the surface resistivity and electromagnetic interference shielding effectiveness for the electrical conductivity were also examined. It was observed that the composite with Cr-20-10 showed better thermal stability and lower resistivity in comparison to the others because, as the cryomilling time and frequency increased the distribution, dispersion and surface area also increased. Consequently, a better interaction between CNTs and PMMA took place.

## 1. Introduction

Recently, conductive polymer composites have gained a substantial amount of attention and have unlocked new opportunities, especially in the field of smart electronic devices [1,2]. Because of the low processing cost and fluctuating conductive performance, conductive polymer composites can be used in various applications, including electromagnetic interference (EMI) shielding, anti-static materials, and sensors [3,4,5]. EMI shielding is a promising technique to augment the performance and endurance of electronic devices. Traditionally, metals, graphite, and conductive polymers are utilized for EMI shielding. Nevertheless, polymer-based shielding materials are extremely convenient due to their ease of synthesis, very light weight and flexibility, and low production cost. Primarily, the aspect ratio and intrinsic conductivity of the filler material are the main factors which affect the efficiency of the EMI shielding material. The significant value of the conductivity of the insulating polymer matrix can be enhanced by incorporating conductive fillers. However, the increment of the conductivity depends on the type and amount of filler material [6,7] as well as the limiting amount at which the conductivity of the composite reaches its critical value, known as the percolation threshold [8].

Numerous reports have been published related to the reinforcement of various fillers including conductive polymers, carbon black, graphene, metal particles, and carbon nanotubes (CNTs) [9,10,11,12]. Among them, CNTs are the most widely used conductive fillers due to their extraordinary electronic and mechanical properties. Furthermore, compared to the other fillers, a small amount of CNTs is sufficient to enhance the thermal, mechanical, and electronic properties, making the resulting composite cost-efficient [13]. Since the discovery of CNTs by Iijima, plentiful research has been performed to boost the properties of conventional polymer matrices by incorporating and modifying the properties of CNTs. For instance, Lee *et al.* mixed CNTs with bio-degradable polymers, starch and chitosan, and reduced their surface resistivity up to four times compared to pure CNTs [14]. Mao *et al.* synthesized a three-dimensional network of MnO_2_ microspheres inner-coated with CNTs, which increased the electronic and ionic conductivities [15]. However, as-grown CNTs possess many defects, including non-uniformity and impurities. Additionally, because of their high aspect ratio, uniform dispersion of CNTs into the matrix is a major hindrance in accomplishing the full potential of CNT-based composites. Therefore, to surmount these issues, countless chemical and mechanical methods have been applied to amend the quality and properties of the resulting composites. Gomez *et al.* used a two-step process to modify the CNTs by removing the metal catalyst particles [16]. In that process, CNTs were first heated in a microwave followed by high-temperature chlorination, which reduced the amount of embedded metal catalysts, along with a very small degree of oxidation of CNTs. Yang *et al.* synthesized γ-FeNi decorated CNT/epoxy composites and observed that the CNTs are uniformly dispersed into the epoxy matrix. As a result, the electrical conductivity of the composites increased, and the composites exhibited semi-conducting behavior [17]. Grunlan *et al.* used functionalized SWNTs with a stabilizer gum arabic followed by reinforcement into a PVAc emulsion and found that the percolation threshold decreased up to twofold compared to non-functionalized carbon black-based PVAc composites [18]. Singh and his colleagues improved the electrical conductivity of polyimide films from 6.67 × 10^−18^ S·cm^−1^ (pure films) to 0.94 S·cm^−1^ by incorporating acid-treated MWNTs [19]. On the other hand, various mechanical methods such as shaking, ultrasonication, ball milling, a nanomizer, and a high-pressure jet mill are also practiced to achieve optimized dispersion of CNTs [20,21,22]. Yi *et al.* enhanced the dispersion of nanoparticles (MWCNTs or Al_2_O_3_) into the PDMS matrix by using a shaker at speeds varying from 1200 to 2200 rpm and found that uniform distribution of the nanoparticles increases and the size of agglomerates decreases at shaking speed [21].

High-energy ball milling is considered one of the most promising techniques to disperse CNTs into a matrix. It is a single-step, cost-efficient, industrial, eco-friendly, and dry method to uniformly disperse CNTs, which is widely used for CNT-metal matrix composites [23,24]. Furthermore, several studies have demonstrated that CNTs can be reformed with a short length, open ends, and smaller diameter by peeling the walls and using curved, less entangled, or exfoliated forms [25,26,27,28]. Nevertheless, sometimes, the original properties of the CNTs are hampered, impaired sp^2^ carbon is generated, or cold-welding, distortion, and fragmentation occur because of the severe milling conditions. The milling speed, milling time, ball-to-powder ratio, and milling temperature are the foremost aspects that affect the ultimate product. To avoid heat generation problems, cryomilling, by which the milling chamber is continuously cooled in the presence of liquid nitrogen where a very low temperature (−180 °C) is maintained, is used. It was found that, because of the cryogenic conditions, the processing time can be reduced, and effective embrittlement and proper dispersion of CNTs can also be achieved.

Although there are many articles related to the ball-milling of CNTs, detailed studies related to the effects of the cryomilling conditions on the properties of CNTs have yet to be carried out. Herein, we prepared poly(methyl methacrylate) (PMMA)/CNT composites, as PMMA is a very cost-effective, easily fabricated, and environmentally stable thermoplastic polymer [29]. The CNTs were modified by applying cryomilling at different frequencies for various lengths of time. To avoid hampering of the CNT structure and properties, the processing time was reduced. Field emission scanning electron microscope (FESEM) and high-resolution transmission electron microscope (HR-TEM) analyses were performed to observe the generated morphological changes. Raman analysis was carried out to evaluate the changes of the crystallinity, and the specific surface area was calculated by using the Brunauer–Emmett–Teller (BET) technique. The thermal stability was examined by thermogravimetric analysis (TGA). The electrical conductivity of the composites was analyzed by measuring the surface resistivity, and the EMI shielding effectiveness (EMI SE) was also investigated.

## 2. Materials and Methods

### 2.1. Required Materials

Raw multiwalled CNTs, which were purchased from Hanhwa Nanotech Co. Ltd., Incheon, Korea, were synthesized by a CVD method with 95% purity. The diameter of the CNTs was 10–15 nm with a 10–20-nm tube length. *N*,*N*-Dimethylformamide (DMF) was purchased from Daejung Chemicals, Siheung-si, Korea. PMMA (*M*_w_ = 120,000, ACS grade) was purchased from Sigma-Aldrich Co., Yongin-si, Korea.

### 2.2. Cryomilling of CNTs

To acquire the cryomilled CNTs, 1 g of raw multiwalled CNTs was placed into a cylindrical (38-mm diameter) stainless steel cryomilling chamber in the presence of a stainless steel ball with a diameter of 25 mm. The weight ratio of the ball to CNTs was 500:1. The cryomilling chamber was designed to oscillate horizontally in the presence of liquid nitrogen at different frequencies for different times. The CNTs prepared at various frequencies and times evaluated in this study are raw CNTs (RCNTs), CNTs cryomilled for 10 min at 10 Hz (Cr-10-10), CNTs cryomilled for 10 min at 20 Hz (Cr-20-10), CNTs cryomilled for 10 min at 25 Hz (Cr-25-10), CNTs cryomilled for 5 min at 20 Hz (Cr-20-5), and CNTs cryomilled for 15 min at 20 Hz (Cr-20-15).

### 2.3. Preparation of PMMA/CNT Composites

PMMA/CNT composites were prepared by dispersing 1 wt % CNTs (each sample) into 150 mL of DMF by ultra-sonicating for 60 min. Later, 4.95 g of PMMA powder were dissolved into the CNT solution by stirring at room temperature for 60 min. The resulting mixture was degassed for 30 min at 60 °C, and then poured into Teflon molds and maintained at 80 °C overnight to obtain the PMMA/CNT film.

### 2.4. Characterization

The morphology of the CNTs was examined by using a field emission scanning electron microscope (FE-SEM) and high-resolution transmission electron microscope (HR-TEM). The samples were prepared by dissolving 0.001 g of the CNTs into ethanol followed by sonication for 1 h. To analyze the crystallinity and defects of the cryomilled CNTs, Raman spectroscopy was performed using a Jasco Raman spectrometer (Tokyo, Japan) equipped with a charge-coupled device (CCD) detector at wavelengths ranging from 100 to 3200 cm^−1^. The specific surface area and average pore volume were calculated by employing the Brunauer–Emmett–Teller technique using BELmax 00131 equipment (BELSORP, Tokyo, Japan). Prior to the analysis, the samples were heated to 250 °C for 7 h. The thermal stability of the composites was investigated using a thermogravimetric analyzer (Q5000 IR/SDT Q600-TA-Instrument, Seoul, Korea) programmed at a heating rate of 10 °C/min in the presence of nitrogen gas. The surface resistivity was measured using a low resistance meter equipped with an ESP probe and the prepared specimens according to the ASTM D991 method. The EMI SE values were calculated using a E5071C Agilent Network Analyzer (ZVA4, Rohde & Schwarz, Munich, Germany) in the measurement range of 100 MHz to 1.5 GHz.

## 3. Results and Discussion

### 3.1. FESEM

**Figure 1:**
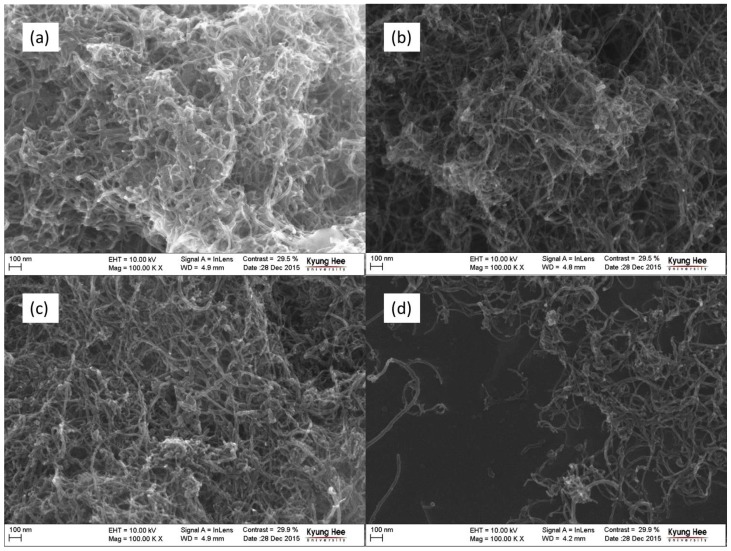
Field electron scanning electron microscope (FE-SEM) images of carbon nanotubes (CNTs): (**a**) raw CNT (RCNT); (**b**) Cr-10-10; (**c**) Cr-20-10; and (**d**) Cr-25-10. The sample Cr-25-10 shows less agglomeration, more dispersion, shorter tube length, and more amorphous carbon in comparison to the others.

**Figure 2:**
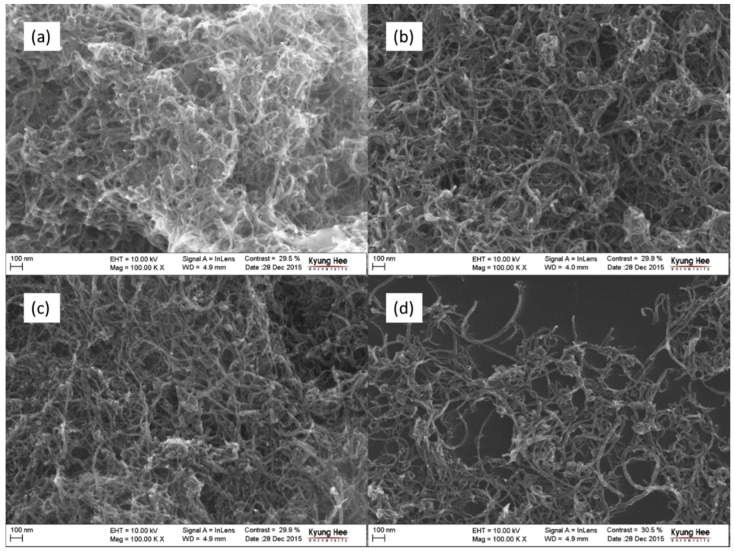
FE-SEM images of CNTs: (**a**) RCNT; (**b**) Cr-20-5; (**c**) Cr-20-10; and (**d**) Cr-20-15. The sample Cr-20-15 shows less agglomeration, more dispersion, shorter tube length, and more amorphous carbon in comparison to the others.

**Figure 3:**
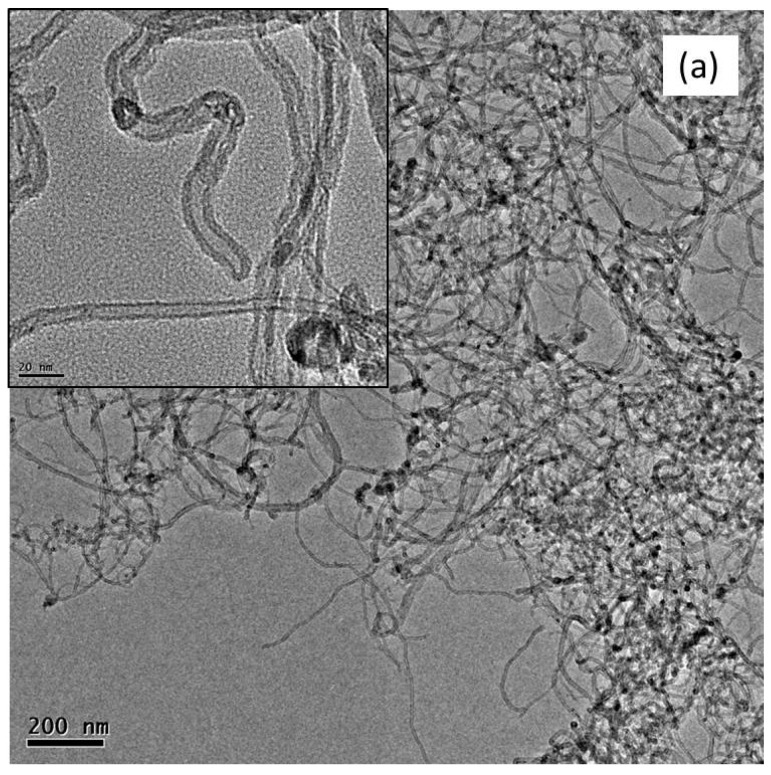

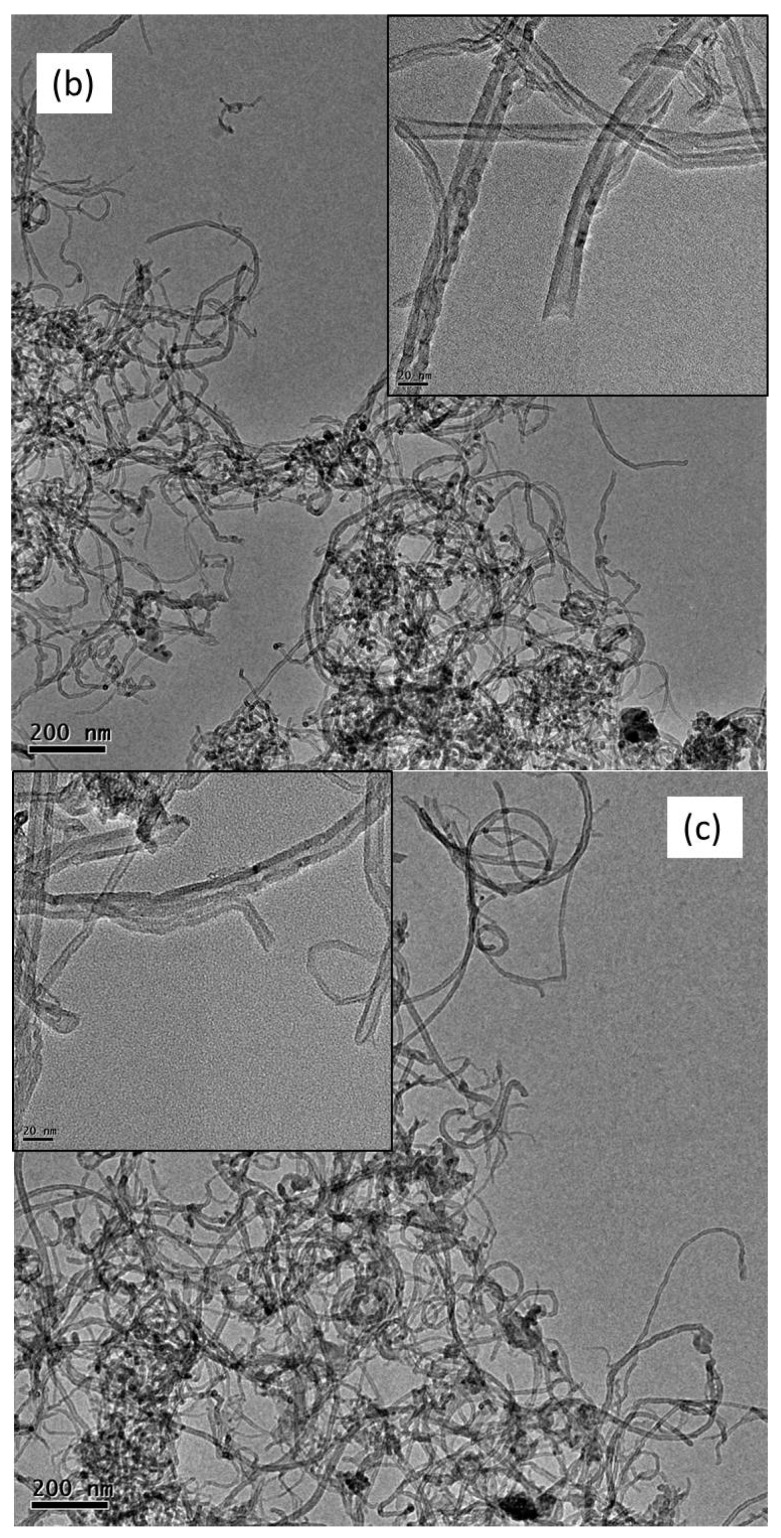
Transmission electron microscope (TEM) images of (**a**) RCNT; (**b**) Cr-25-10; and (**c**) Cr-20-15. For sample RCNT, tubes were highly agglomerated with intact wall and closed end, whereas, for Cr-25-10 and Cr-20-15, tubes were less agglomerated, shorter, and open along with amorphous carbon debris.

Figure 1 and Figure 2 show the FE-SEM images of the raw and cryomilled CNTs. The morphology of the cryomilled CNTs obtained at a constant time (RCNT, Cr-10-10, Cr-20-10, and Cr-25-10) and constant frequency (RCNT, Cr-20-5, Cr-20-10, and Cr-20-15) is shown in Figure 1 and Figure 2, respectively. As can be observed in the images, the raw CNTs (a) show a high degree of agglomeration and are densely entangled. Conversely, in the case of cryomilled CNTs (Figure 1b–d), relatively less agglomeration or less entanglement is observed. Furthermore, the length of the cryomilled CNTs became shorter than that of the raw CNTs as a result of the brittle nature of the CNTs obtained under cryogenic conditions. Owing to the reduced entanglement, the dispersibility of the cryomilled CNTs increased gradually with increasing frequency and time. Among the CNTs cryomilled at frequencies of 10, 20, and 25 Hz, Cr-25-10 showed better dispersion with a less webby nature and shorter length. Moreover, this shows that amorphous carbon debris is generated by crushing of the tubes between the ball and chamber walls. A similar trend is observed in the CNTs cryomilled at a constant frequency (Figure 2) at various times. Among all of the samples, Cr-20-15 shows better dispersion, as well as a less dense morphology with a shorter tube length. The FE-SEM results were further validated by the TEM images shown in Figure 3.

### 3.2. TEM

Figure 3 shows the TEM images of the RCNT, Cr-25-10, and Cr-20-15 samples. Similar to the FE-SEM images, it is seen that the raw CNTs (a) exhibit dense packing. Furthermore, the tube ends were found to be closed. In addition, the tube walls were also intact and continuous without any kind of peeling or distortions. Conversely, the cryomilled CNTs (b and c) show better dispersibility and less agglomeration. Figure 3b,c show TEM images of Cr-25-10 and Cr-20-15, respectively. As shown in the inset, the tube ends are open or partially open. Moreover, in some tubes, the outer walls are peeled off because of the high-energy cryomilling; consequently, the wall diameter decreased compared to the raw CNTs. Because of this, in some places, amorphous carbon debris is also observed. In addition, other defects, including buckling, raptures, and discontinuities, were also detected.

### 3.3. Raman Spectroscopy

**Figure 4:**
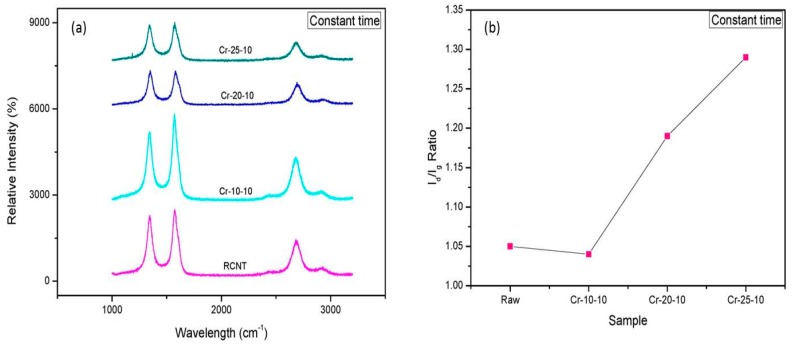
(**a**) Raman spectra; and (**b**) *I*_d_/*I*_g_ ratios of RCNT, Cr-10-10, Cr-20-10, and Cr-25-10. The *I*_d_/*I*_g_ graph shows that defects in CNTs increase as the cryomilling speed increases.

**Figure 5:**
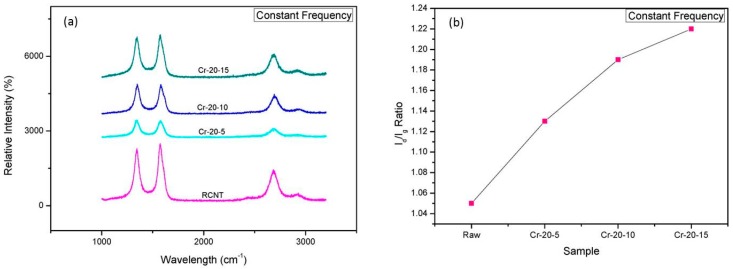
(**a**) Raman spectra; and (**b**) *I*_d_/*I*_g_ ratios of RCNT, Cr-20-5, Cr-20-10, and Cr-20-15. The *I*_d_/*I*_g_ graph shows that defects in CNTs increase as the cryomilling time increases.

Raman spectroscopy was performed to study the effects of the cryomilling conditions on the structure and crystallinity of the nanotubes. Figure 4a and Figure 5a compare the Raman spectra of the CNTs cryomilled at a constant time and frequency, respectively. As observed from the spectra, all CNTs exhibit the characteristic peaks of CNTs. The peak around 1340 cm^−1^ is ascribed to the defects generated because of the sp^2^ carbons on the CNT walls and is known as the D-band. The peak around 1570 cm^−1^, which is responsible for the in-plane vibrations of sp^3^ carbons, represents the crystallinity of graphitic structure and is known as the G-band. One more peak at around 2680 cm^−1^ was also obtained, which is known as the G’-band. It can be seen clearly from Figure 4a and Figure 5a that all the peaks shift towards higher wavelengths. The D-band shifts from 1344 to 1347 cm^−1^, the G-band shifts from 1572 to 1575 cm^−1^, and the G’-band shifts from 2682 to 2686 cm^−1^. This shift of the position and intensity denotes that changes occurred in the CNTs after cryomilling [30]. It can be assumed that the decreased number of CNT walls is responsible for the decrement of the peak intensity with increasing cryomilling frequency and time [26]. A qualitative and quantitative analysis of defects can be performed by calculating the *I*_d_/*I*_g_ ratio (*I*_d_/*I*_g_), which is basically the ratio of the intensity of structural defects to the intensity of graphitization. Figure 4b and Figure 5b display the *I*_d_/*I*_g_ ratios of the corresponding cryomilled CNTs. The *I*_d_/*I*_g_ ratios for RCNT, Cr-10-10, Cr-20-10, and Cr-25-10 are 1.05, 1.04, 1.19, and 1.29, respectively, while, for RCNT, Cr-20-5, Cr-20-10, and Cr-20-15, *I*_d_/*I*_g_ ratios are 1.05, 1.13, 1.19, and 1.22, respectively. The *I*_d_/*I*_g_ ratios of RCNT and Cr-10-10 are almost similar because 10 Hz was not sufficient to affect the densely packed bundle of CNTs; hence, fewer defects were generated. Since the frequency and the time increased, the *I*_d_/*I*_g_ ratio also increased. An increased *I*_d_/*I*_g_ ratio provided validation of the applied physical forces and generated defects, disruptions, disorders, and amorphous carbon due to the harsh cryomilling conditions [27].

### 3.4. BET Analysis

**Figure 6:**
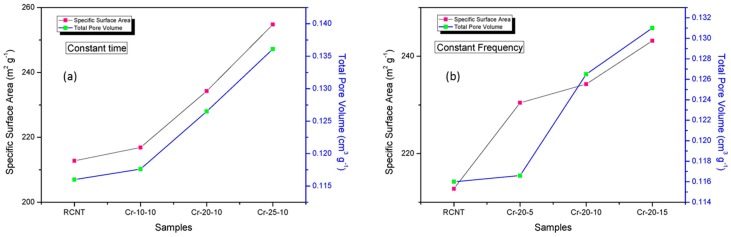
Specific surface area and total pore volume of the (**a**) RCNT, Cr-10-10, Cr-20-10, and Cr-25-10; and (**b**) RCNT, Cr-20-5, Cr-20-10, and Cr-20-15 samples. The samples Cr-25-10 and Cr-20-15 showed the highest specific surface area and highest average pore volume.

The specific surface area and micropore volume of the CNTs obtained at (a) different frequencies and (b) different times were analyzed via BET analysis (Figure 6). It is evident from the figure that the specific surface area and the micro pore volume increase with increasing cryomilling speed and time. The specific surface area increased from 212 (for RCNT) to 254.78 m^2^·g^−1^ (for Cr-25-10) and 243.16 m^2^·g^−1^ (for Cr-20-15). In addition, the total pore volume of the starting material (RCNT) was 0.116 cm^3^·g^−1^, which increased to 0.136 (for Cr-25-10) and 0.131 cm^3^·g^−1^ (for Cr-20-10). As mentioned above, due to the harsh conditions of cryomilling, the quantity of short and broken tubes with open tips and ruptured side walls increased gradually [31]. Additionally, bucking also occurred, and amorphous carbon debris was observed. Consequently, the related specific surface area and pore volume also increased.

### 3.5. Thermal Analysis

**Figure 7:**
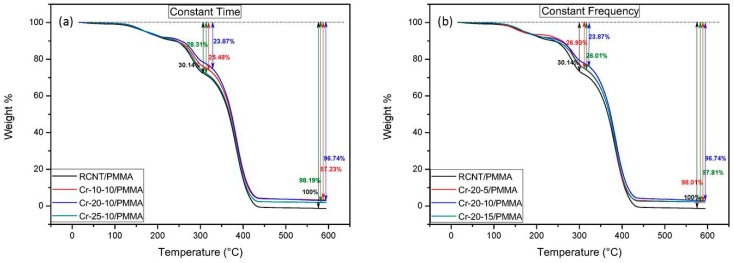
Thermograms of the different CNT/poly(methyl methacrylate) (PMMA) composites: (**a**) RCNT, Cr-10-10, Cr-20-10, and Cr-25-10; and (**b**) RCNT, Cr-20-5, Cr-20-10, and Cr-20-15. Among all, the sample Cr-20-10 is the most stable.

Comparison of the thermal properties of the different CNT/PMMA composites was performed via TGA, and the thermograms are shown in Figure 7. It can be clearly seen that all of the samples followed a three-step degradation process. The first step (between 50 and 160 °C) takes place due to the evaporation of physically adsorbed moisture on the surface of the composite [32]. Degeneration in the side chains and unsaturated end groups of PMMA is responsible for the second step (between 180 and 290 °C) [33]. Finally, the third step (300 °C onwards) occurs because of degradation of the main polymer backbone. For all of the composites, major thermal degradation occurs between 300 and 400 °C. However, the rate of thermal degradation for all of the composites is different, which means that the weight residues are also different. The highest weight loss (100%) was obtained for the RCNT/PMMA composites, while Cr-20-10/PMMA showed the lowest weight loss of 96.74% in both cases (constant time and constant frequency). This significant difference of the thermal stability of the composites demonstrates proper dispersion of the Cr-20-10 CNTs into the PMMA matrix, which offers stability during thermal degradation by restricting the mobility of the polymer chains and acting as a heat barrier. The weight residues for Cr-10-10, Cr-25-10, Cr-20-5, and Cr-20-15 are similar. The reason behind this is that, at lower frequencies and times, agglomeration of the CNTs occurs. Alternatively, at higher frequencies and times, deformation of the CNT structure is higher compared to the others. Additionally, the amount of amorphous carbon also increases due to the increased time and speed of continuous collisions between the ball and cryomilling container wall. Consequently, these amorphous carbon particles and short-length CNTs are less likely to restrict the mobility of the segments of the polymer.

### 3.6. Electrical Conductivity

**Figure 8:**
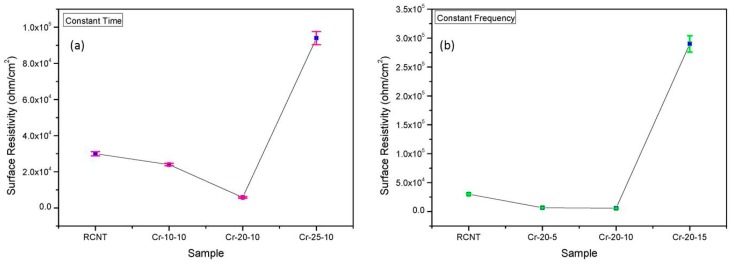
Surface resistivity of the different CNT/PMMA composites: (**a**) RCNT, Cr-10-10, Cr-20-10, and Cr-25-10; and (**b**) RCNT, Cr-20-5, Cr-20-10, and Cr-20-15. The sample Cr-20-10 shows higher conductivity in comparison to the others due to a better dispersion and proper conductive network.

The electrical properties of the different CNT/PMMA composites were examined by using a low resistance meter, and the data is presented in Figure 8a,b. It can be seen in Figure 8a that the surface resistivity decreases with increasing cryomilling frequency up to 20 Hz. The surface resistivities of the RCNT/PMMA, Cr-10-10/PMMA, and Cr-20-10/PMMA composites are 3 × 10^4^, 2.4 × 10^4^, and 5.8 × 10^3^ ohm/cm^2^, respectively. Since it is known that RCNTs are likely to be present in a bundle form due to the Vander Waal interactions, as cryomilling is applied, the CNTs tend to separate and align arbitrarily into the polymer matrix [34]. Hence, uniform dispersion of the CNTs while maintaining a high aspect ratio can be optimized. In the case of Cr-20-10, the size of agglomerates and the entanglement of CNTs decrease due to the increased processing speed. Consequently, the CNTs are well dispersed and well distributed into the matrix, and a proper interconnecting conductive network is formed that leads to a better conductive mechanism such as hoping and tunneling into the polymer matrix [35]. Therefore, the conductivity increases and the surface resistivity decreases (by 50%). However, for the Cr-25-10/PMMA composite, the surface resistivity is the highest with a value of 9.4 × 10^4^ ohm/cm^2^. The reason for this result is that in the presence of liquid nitrogen, the CNTs become heavily brittle and fragile such that a high cryomilling speed develops higher deformations in the CNT structure, consequently lowering the aspect ratio. It is predicted that, because of the low aspect ratio, a continuous conductive network of CNTs is not formed, resulting in a higher resistivity. Further, Figure 8b shows the resistivity of the different CNT/PMMA composites cryomilled for different times at a frequency of 20 Hz. A similar trend is observed as the surface resistivity decreases as milling time increases. The surface resistivity of the Cr-20-5/PMMA composite is 6.6 × 10^3^ ohm/cm^2^, which is slightly higher than that of the Cr-20-10/PMMA composite. The Cr-20-15/PMMA composite shows the highest resistivity of 2.9 × 10^5^ ohm/cm^2^. The reason for this result is believed to be the prolonged milling time of the CNTs under a harsh cryomilling environment, which decreases the aspect ratio of CNTs, resulting in the lack of a continuous network.

### 3.7. EMI Shielding Effectiveness (EMI SE)

The attenuation capability of the shielding material to propagate electromagnetic waves is acknowledged as the EMI shielding effectiveness (EMI SE). It is basically the ratio between the incident (*P*_i_) and outgoing powers (*P*_o_) of the wave [36] and is calculated as follows:

SE (dB) = −10 log_10_ (*P*_i_/*P*_o_) . (1)

Additionally, the total SE (SE_T_) for the incident electromagnetic wave is expressed as the sum of SE due to absorption (SE_A_), reflection (SE_R_), and multiple reflections (SE_M_), as shown below [37]:

SE_T_ = SE_A_ + SE_R_ + SE_M_ . (2)

**Figure 9:**
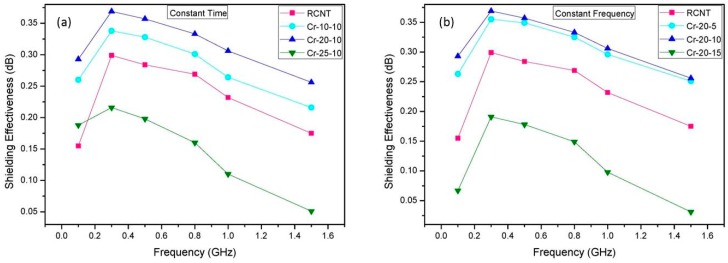
Electromagnetic interference (EMI) shielding effectiveness (SE) data of the different CNT/PMMA composites: (**a**) RCNT, Cr-10-10, Cr-20-10, and Cr-25-10; and (**b**) RCNT, Cr-20-5, Cr-20-10, and Cr-20-15. The sample Cr-20-10 shows higher EMI SE in comparison to the others due to a better dispersion and proper conductive network.

In order to analyze the effect of the cryomilling speed and time on the EMI SE, shielding effectiveness tests were conducted. Figure 9 exhibits the SE of the different CNTs/PMMA composites within a frequency range of 1 MHz to 1.5 GHz. It is clearly noticeable that the pattern of the different CNT/PMMA EMI SE is analogous to the surface resistivity pattern. In the case of the varied frequency (Figure 9a), sample Cr-20-10/PMMA displays the highest EMI SE, while Cr-25-10/PMMA shows the lowest EMI SE. Likewise, in Figure 9b, Cr-20-10/PMMA shows the highest EMI SE, whereas Cr-20-15/PMMA exhibits the lowest EMI SE. As mentioned above, in the case of Cr-20-10, increased cryomilling time leads to less bundled CNTs, inducing better dispersion and distribution of CNTs into the polymer matrix. Therefore, well dispersed CNTs make a proper interconnected network that leads to better conductivity [35]. Since it is known that intrinsic conductivity of the filler material affects the EMI SE of the composite, the sample with Cr-20-10 exhibits better EMI SE in comparison with the others. On the other hand, Cr-25-10/PMMA and Cr-20-15/PMMA EMI SE possess the lowest EMI SE. The presumed reasoning behind this outcome is that the increased cryomilling speed and time engender the deformed CNTs along with a low aspect ratio, which consequently diminishes the conductivity compared to the other samples, and the EMI SE is directly affected by conductivity.

## 4. Conclusions

In this study, the effects of different cryomilling conditions on the properties of CNTs were investigated. Six samples of CNTs were prepared: RCNT, Cr-10-10, Cr-20-10, Cr-25-10, Cr-20-5, and Cr-20-15. PMMA/CNT composites were synthesized by incorporating these different CNTs into the PMMA matrix. Morphological studies were performed using FESEM and TEM, and qualitative analysis was carried out through Raman spectroscopy. It was found that the dispersion increases with increasing cryomilling time and speed. Additionally, the length of the CNTs became short, and different kinds of defects occurred due to the harsh conditions of cryomilling. The specific surface area and average pore volume increased with increasing cryomilling time and frequency. Subsequently, thermal stability, surface resistivity, and EMI SE were also measured, and it was observed that composites with Cr-20-10 exhibited better thermal stability and lower resistivity in comparison to the others, because, with increased cryomilling frequency and time, more uniform dispersion took place. Hence, a proper network of CNTs was formed, which offered stability during thermal degradation and provided a better conductive mechanism during surface resistivity tests. However, for composites with Cr-25-10 and Cr-20-15, the thermal stability and conductivity decreased, because the increased frequency and time of the cryomilling gave rise to the deformation of the basic structure of the CNTs along with an increased amount of amorphous carbon. As a result, a continuous network cannot form; consequently, the resistivity increases.
